# Nutritional and biochemical insights into multiple pressure ulcer management: a case report

**DOI:** 10.3389/fnut.2025.1564879

**Published:** 2025-04-24

**Authors:** Alica Hokynková, Hana Paulová, Petr Šín, Marie Nováková, Petr Babula, Andrea Pokorná

**Affiliations:** ^1^Department of Burns and Plastic Surgery, University Hospital, Brno, Czechia; ^2^Faculty of Medicine, Masaryk University, Brno, Czechia; ^3^Department of Biochemistry, Faculty of Medicine, Masaryk University, Brno, Czechia; ^4^Department of Physiology, Faculty of Medicine, Masaryk University, Brno, Czechia; ^5^Department of Health Sciences, Faculty of Medicine, Masaryk University, Brno, Czechia

**Keywords:** C-reactive protein, oxidative stress, prealbumin, pressure ulcer, case report, nutritional status, multistage surgery, 8-hydroxy-2′-deoxyguanosine

## Abstract

This case report presents a unique study focused on relationship between nutritional parameters, C-reactive protein (CRP), and the marker of oxidative stress 8-hydroxy-2′-deoxyguanosine (8-OHdG), in 61-year-old male paraplegic patient with multiple pressure ulcers. A multidisciplinary approach was essential for the timing of the successful reconstruction process. The patient had a history of paraplegia since 1999 due to a skiing accident with fractures of thoracic vertebrae (Th6–Th9), requiring spinal surgery. His medical background includes acute pancreatitis with biliary tract revisions, partial pancreatic resection, acute respiratory failure with tracheostomy, renal failure treated with hemodialysis, bronchopneumonia, pseudomembranous colitis, and hyperuricemia. The patient also underwent multiple surgical interventions, including treatment for ulnar nerve paresis, cholecystectomy, and multiple pressure ulcer reconstructions. The study describes the relationship between selected biochemical parameters and overall clinical status in a paraplegic patient with three deep pressure ulcers located in the left-sided ischial, trochanteric, and sacral regions during their multistage surgical therapy. The reconstructive procedure and collection of biological samples for determination of selected biochemical parameters were performed according to the same schedule: debridement of pressure ulcers at the beginning of the particular hospitalization and on the day of surgical reconstruction. The patient’s severe condition was accompanied by decreased levels of both 8-OHdG and selected nutritional parameters (albumin, prealbumin, and total protein) and increased CRP levels at the beginning of the treatment process. The evaluation of the dynamics of the measured parameters during the gradual improvement of the patient’s condition and the multistage reconstruction of pressure ulcers in six hospitalizations over a period of 17 months was continued resulting in the healing of all pressure ulcers. This case highlights the crucial importance of investigating selected biochemical parameters, with emphasis on 8-OHdG, and nutritional parameters, for the timing of surgical strategy and comprehensive therapy in patients with multiple pressure ulcers and severe, complex medical histories.

## Introduction

Extensive and multiple deep pressure ulcers (PUs) represent a serious medical and socio-economic problems, especially in immobile patients (1). Their healing is negatively influenced by numerous factors, such as comorbidities, duration and number of hard-to-heal wounds, their local status (e.g., category, size, biofilm, osteomyelitis), and patients’ compliance and motivation (2). The success of surgical interventions depends, among other things, on the actual health status of the patient, monitored by a number of laboratory parameters, including nutritional ones (2). Another factor affecting the course of surgical therapy and subsequent healing is oxidative stress (3). One of the important markers of oxidative stress is 8-hydroxy-2′-deoxyguanosine (8-OHdG), an indicator of oxidative DNA damage (4). Although DNA damage can be assessed by various approaches (e.g., comet assay (5)) the most used parameter is 8-OHdG. It is stable molecule, freely filterable into the urine, where it can be subsequently determined.

This case report presents the dynamics of selected nutritional, inflammatory, and biochemical parameters in relation to planned multistage surgical therapy in a paraplegic patient with multiple PUs over a period of 17 months.

## Case description

**Figure 1:**
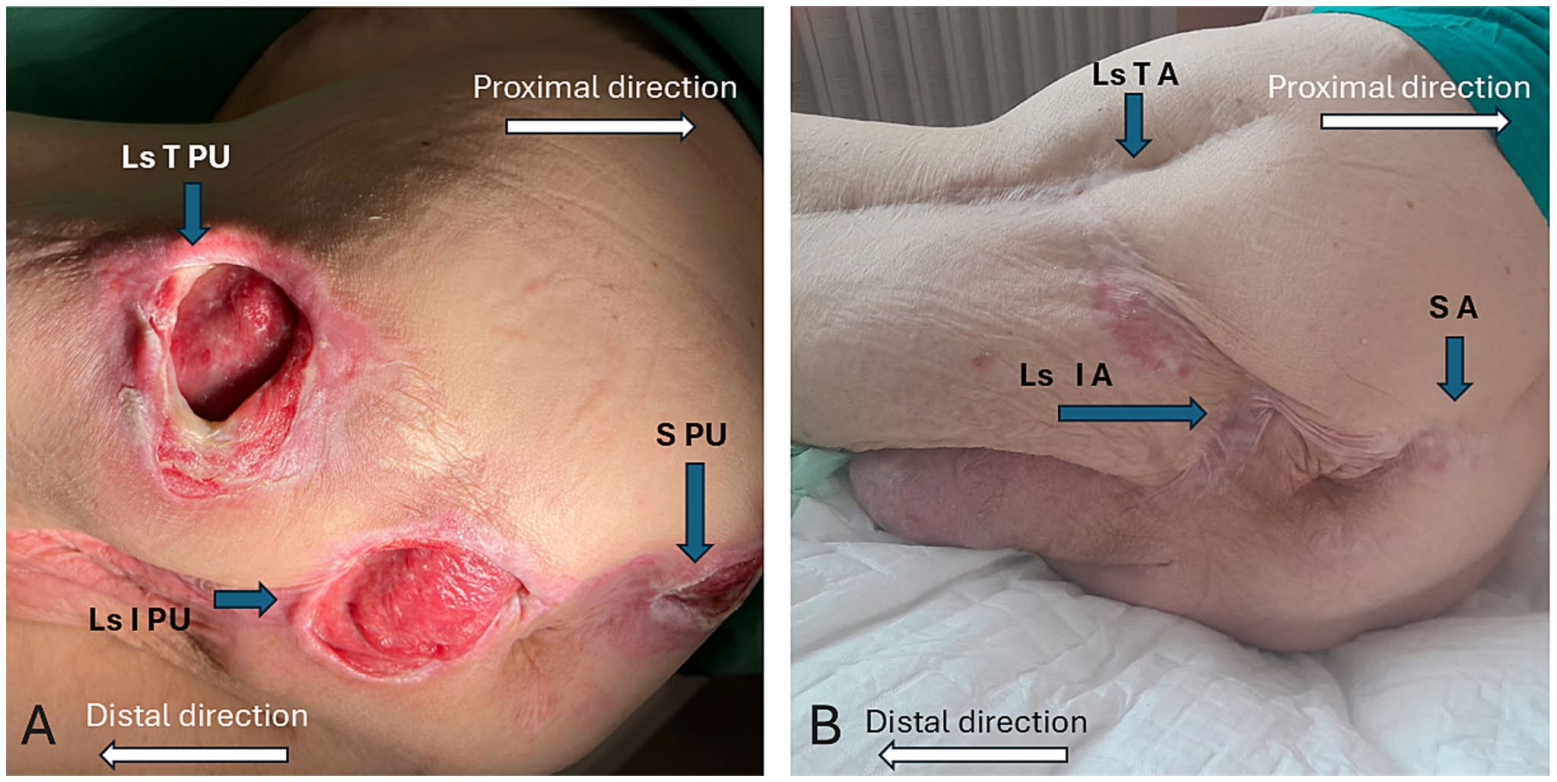
**(A)** Localization and pressure ulcers status before treatment (left-sided trochanteric PU - Ls T PU, left-sided ischial PU – Ls I PU, sacral PU – S PU) and **(B)** Healed pressure ulcers areas after treatment (left-sided trochanteric area – Ls T A, left-sided ischial area – Ls I A, sacral area – S A).

A 61-year-old male paraplegic patient (at the time of the first admission) with three PUs of deep category located in the left-sided ischial and trochanteric and sacral regions (see Figure 1) was planned for multi-stage reconstructive surgical treatment. The patient has been paraplegic since 1999 due to a skiing accident, which resulted in the fractures of thoracic vertebrae (Th6–Th9) and subsequent spinal surgery. He has a complex medical history, including acute pancreatitis requiring biliary tract revisions and partial pancreatic resection, acute respiratory failure with tracheostomy, and renal failure treated with hemodialysis. Additional complications include left-sided bronchopneumonia, pseudomembranous colitis, and hyperuricemia. Surgical interventions include treatment for ulnar nerve paresis, cholecystectomy with biliary tract revisions, and multiple PUs surgeries over several years. This history highlights the patient’s extensive medical challenges and the need for multidisciplinary care. This case report describes the course of comprehensive therapy and management of three extensive and deep PUs during repeated hospitalizations (H1–H6) between February 2022 and July 2023, with a focus on selected biochemical and nutritional parameters.

The patient’s overall condition fluctuated over time, and his clinical status corresponded with the local wound symptomatology. During H1–H3, the patient suffered from infection and renal insufficiency. These infections included signs of local infection with polymicrobial colonization of the PUs, systemic infection with positive blood cultures during H1, and a urinary tract infection in H2. Logically, the wound extents were the largest during this period. Moreover, some extrinsic factors influenced the course of therapy, such as an intestinal infection (*Clostridium difficile* colitis) in H4. The improved patient’s status during the H5-H6 hospitalizations allowed for the finalization of reconstructive surgeries, which were always planned according to the actual clinical condition and local wound symptomatology. Clinical data of the patient and wound status during the six hospitalizations are summarized in Table 1.

**Table 1:** Clinical data of the patient.

CLINICAL DATA						
Pressure ulcer location	H1	H2	H3	H4	H5	H6
Ischial left side						
Category	IV	IV	IV	IV	IV	IV
Size (length, width, pocket)*	9x8cm	9x6cm	8x5cm	7x3cm pocket:10x3cm	5x4cm pocket:7x6cm	6x4cm pocket:9x9cm
Debridement	Day 0: YES Day 14: YES partial resection of osteolytic ischial tuber	Day 0: YES	Day 0: YES	Day 0: YES	Day 0: YES	Day 0: YES
Reconstruction	NO	NO	Day 0: YES skin graft	Day 7: YES, local flap	NO	Day 7: suture
Local complications	Wound colonization *Enterococcus faecium VRE*	Polymicrobial colonization	*Pseudomonas aeruginosa*	*Proteus mirabilis*, *Acinetobacter baumanii*	Polymicrobial colonization	*Acinetobacter baumanii*
Trochanteric left side						
Category	IV	IV	IV	Healed	Healed	Healed
Size (length, width, pocket)*	5x5cm deep pocket 10x8cm	3x wound dehiscence	6x3cm	NA	NA	NA
Debridement	Day 0: YES Day 14: YES Partial resection of osteolytic caput femoris	Day 0: YES	Day 0: YES	NA	NA	NA
Reconstruction	Day 14: YES biceps femoris muscle + direct suture	NO	Day 0: YES dorsal thigh flap	NA	NA	NA
Local complications	Osteomyelitis, *Enterococcus faecium VRE,* *Candida glabrata*, Wound dehiscence	Polymicrobial colonization	*Pseudomonas aeruginosa*	NA	NA	NA
Sacral						
Category	IV	IV	IV	IV	IV	Healed
Size (length, width, pocket)*	6x5cm	10x10cm	8x5cm	4x4cm pocket:11x5cm	4x4cm pocket:11x5cm	NA
Debridement	Day 0: YES Day 14: YES partial resection of osteolytic os sacrum	Day 0: YES	Day 0: YES	Day 0: YES	Day 0: YES	NA
Reconstruction	NO	NO	NO	Day 7: YES rotation gluteal fasciocutaneous flap	DAY 0: YES revision, tissue sealant, suture	NA
Local complications	Wound colonization *Enterococcus faecium VRE*, *Proteus mirabilis*	Polymicrobial colonization	NO	Polymicrobial colonization *Candida albicans,* floating flap plasty	Polymicrobial colonization	NA

*Size: There was not always possible to measure depth; NA, Not applicable.

**Figure 2:**
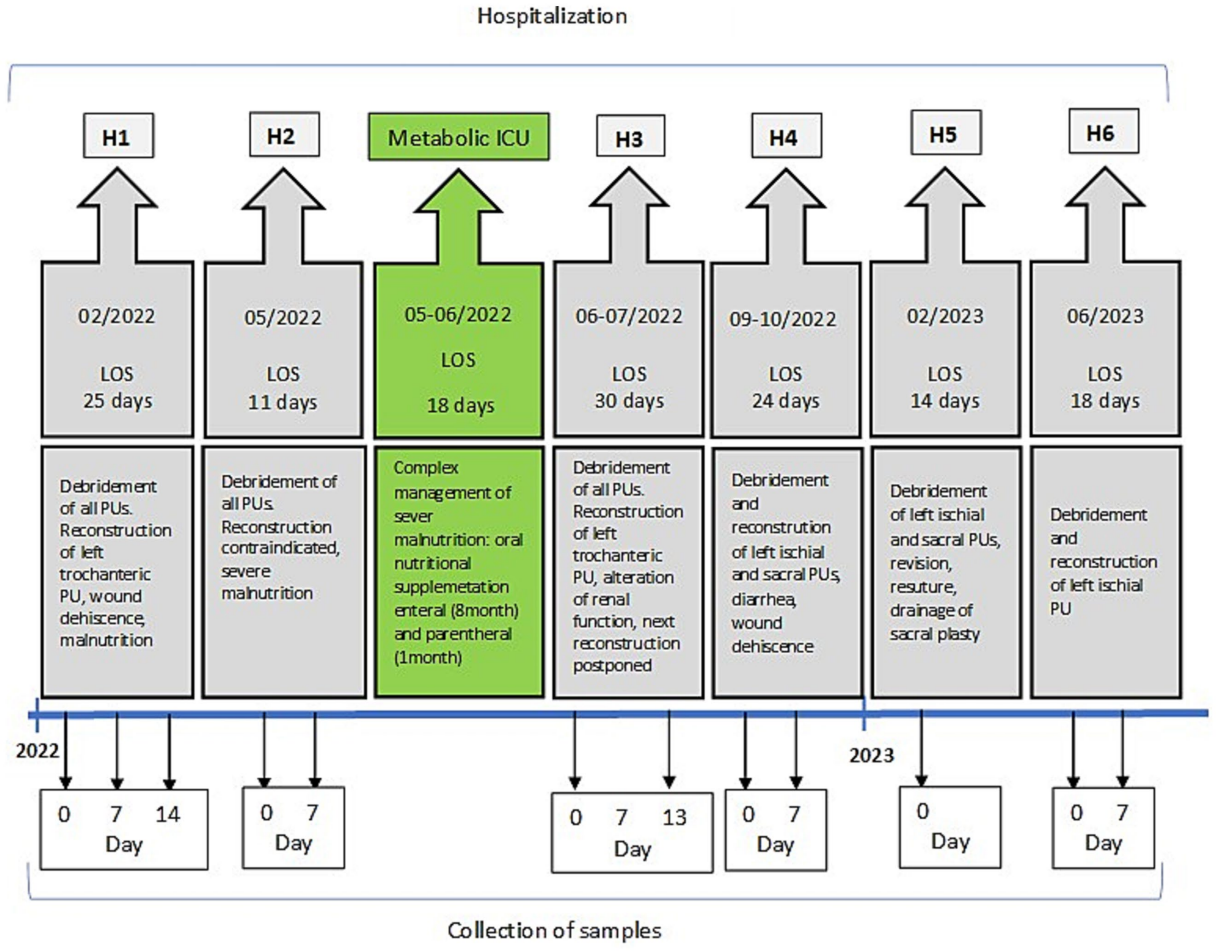
Timeline of the case. More extensive photo documentation and data are available and can be obtained from the main author upon reasonable request.

The clinical status of the patient was characterized by nutritional and biochemical parameters measured from plasma and urine. The samples were collected at the same time points as surgical interventions (if performed) - debridement of PUs at the beginning of the particular hospitalization (Day 0) and further reconstruction (Day 7). Due to the actual clinical situation of the patient, some samples could not be collected at all or were collected on different days of hospitalization. The detailed timeline outlining the progression and key events of the case report is illustrated in Figure 2.

In total, 59 biological samples related to surgical therapy (debridement and reconstruction) were analyzed over a 17-month period. The basic nutritional parameters (prealbumin and albumin) and biochemical parameters (total protein and CRP) were assessed from plasma samples using routine commercial kits (Roche, Switzerland). The oxidative stress parameter 8-OHdG was determined from morning urine samples using a modified LC/MS/MS method (6). The urinary concentrations of 8-OHdG were expressed in ng/mg creatinine. The measured values of all parameters are summarized in Table 2.

**Table 2:** Nutritional and biochemical parameters, daily protein and caloric intake, nutritional therapy.

Nutritional and biochemical parameters							
Parameter	Day	H1	H2	H3	H4	H5	H6
Prealbumin (g/L)							
	Day 0	0.19	0.09	0.24	0.19	0.21	0.19
	Day 7	0.15	0.07	#	0.18	#	0.17
	Day X	0.17^a^	##	0.16^b^	##	##	##
Albumin (g/L)							
	Day 0	29.6	20.8	23.3	26.7	28.6	29.4
	Day 7	27.1	19.5	#	25.6	#	26.9
	Day X	25.3^a^	##	25.2^b^	##	##	##
Total protein (g/L)							
	Day 0	74.0	70.9	79.0	89.8	82.6	78.5
	Day 7	70.2	67.9	#	86.6	#	87.9
	Day X	66.6^a^	##	90.8 ^b^	##	##	##
CRP (mg/L)							
	Day 0	70.1	157.2	44.2	44.2	23.6	20.9
	Day 7	88.8	156.1	#	52.0	#	29.9
	Day X	41.8^a^	##	91.0^b^	##	##	##
8-OHdG (ng/mg crea)							
	Day 0	13.3	10.44	19.66	9.38	7.63	9.8
	Day 7	#	8.04	#	12.63	#	9.41
	Day X	17.07^a^	##	6.67^b^	##	##	##
Daily calculation and nutritional therapy							
Daily protein intake		71g	122g	100g	100g	Not calculated	Not calculated
Daily caloric intake		5020kcal	2253kcal	2340kcal	2190kcal	Not calculated	Not calculated
Nutritional therapy per os		Sipping (Cubitan 2x200ml, Nutridrink compact protein- 2x 200ml/24 h) Fresubin Protein Powder -3x2 spoon /24h	Sipping (Cubitan)- 2x200ml /24 h Fresubin Protein Powder -3x2spoon /24h	Sipping (Cubitan)- 2x200ml /24 h Fresubin Protein Powder -3×2spoon /24h	Sipping (Cubitan)- 2x200ml /24 h Fresubin Protein Powder -3×2spoon /24h	Sipping (Diben drink 1.200ml/24 h, Cubitan 1x200ml/ 24 h)	Sipping (Cubitan)- 2x200ml /24 h Fresubin Protein Powder -3x2spoon/ 24h
Nutritional therapy enteral via nasojejunal tube		None	None	Nutrison Diason Energy HP 250ml/24 h	Diben 500ml/24 h	None	None
Nutritional therapy parenteral via central venous catheter		None	None	All in One bags 910ml/24 h (940 kcal, 60g proteins)	None	None	None

H, Hospitalization; #-The sample was not collected due to patient’s clinical status. ## - The collection of the samples was not planned. Day X – The day not primally planned for the sample collection (^a^Day 14, ^b^Day 13).

Surgical therapy was initiated with the debridement of all PUs during H1. However, it was necessary to respond to the patient’s unsatisfactory condition (signs of malnutrition and infectious complications), which lasted from H1-H3. Therefore, the reconstruction of PUs had to be spread over a longer period of time. In H1, the extirpation of the osteomyelitic femoral head on the left side, along with partial resection of the sacral and ischial tuber bone tissue for the osteolytic process, was performed. A previous flap plasty (done several years ago) above the resected femoral head on the left side was elevated and transponed over the trochanteric PU to allow successful wound closure. The postoperative status was further complicated by the presence of *Vancomycin-resistant Enterococcus faecium (VRE)* in all three PUs. Additionally, the femoral head resecate was accompanied by a positive hemoculture of *Proteus mirabilis*. The patient’s nutritional status began to worsen, and CRP levels increased. Therefore, nutritional support (sipping and high protein diet), conservative local therapy to eradicate biofilm, and prolonged systemic antibiotic therapy were indicated.

During the second hospitalization, further surgical management of multiple PUs was planned. Debridement was performed in all affected areas; however, due to severe malnutrition, reconstructive surgery was contraindicated. A significant deterioration of nutritional status (decrease in prealbumin and albumin) occurred, leading to reopening (dehiscence) of the left trochanteric PU. CRP levels increased significantly, whereas 8-OHdG concentrations simultaneously decreased.

Between the second and third hospitalization, the patient was admitted to the Metabolic ICU for intensive nutritional support and stabilization of nutritional parameters. The patient received enteral nutrition via a nasojejunal tube, which was maintained for a total of 8 months. In parallel, parenteral nutrition using All-in-One bags was administered for 1 month to support metabolic stabilization and optimize nutritional parameters. This comprehensive nutritional intervention aimed to optimize the patient’s metabolic status and ensure an adequate supply of essential nutrients necessary for surgical treatment and recovery at the beginning of H3.

The flap plasty of recurrent trochanteric PU was performed. However, during H3, the patient’s general clinical condition worsened, as demonstrated by an increase in CRP and a decrease in prealbumin and 8-OHdG. Therefore, the next reconstructive surgical procedure was postponed for 2 months. In H4, the flap plasty of the sacral and left side ischial PUs was performed. Although the monitored parameters did not indicate any complications or worsening of health status at the beginning of H4, the patient’s condition deteriorated again. This situation was attributed to an infection with *Clostridium difficile colitis*, suspected to be a hospital acquired infection (7).

Repeated contamination of sutured wounds with stool led to complete dehiscence of the sutured wound above the left ischial area and the release of the rotation flap in the sacral region, known as floating flap plasty. Consequently, reoperation using tissue sealant was necessary. During periods H5-H6, the reconstruction of the remaining PUs was gradually completed.

To provide a detailed overview of the patient’s nutritional intake, daily caloric and protein intake during the H1–H6 period, along with the type of nutritional therapy used, is summarized in Table 2. These values reflect the dynamic changes in metabolic support provided throughout the hospitalization periods. The patient remained under continuous supervision by nutritional specialists from the first to the last hospitalization. Throughout the entire course of treatment, nutritional experts closely monitored the patient’s condition, adjusting the nutritional strategy as needed to ensure optimal metabolic support and recovery.

From the patient’s perspective, the prolonged treatment period and multiple hospitalizations posed significant physical and psychological challenges. The patient reported frustration due to wound recurrence, surgical complications, and prolonged recovery times. However, the gradual improvement of biochemical parameters and clinical condition reinforced trust in the multidisciplinary approach, and the patient expressed high satisfaction with the final surgical outcomes.

## Discussion

In this paper, we provide a comprehensive insight into the dynamic interplay among nutritional status, oxidative stress, and inflammatory markers in the context of multistage surgical reconstruction of multiple PUs in a paraplegic patient. The systemic therapy of patients with multiple PUs must reflect health status in combination with biochemical parameters according to Evidence Based Medicine (8). The nutritional status, as reflected by albumin and prealbumin levels, is essential for the successful continuation of PU reconstruction (9). Although several nutritional biomarkers are known (10), we focused on two routinely used ones: albumin and prealbumin. Among them, prealbumin is more useful for assessing nutritional status due to its shorter half-life (2–3 days *vs.* 19 days for albumin), indicating that it reflects rapid changes in nutritional state (11). However, it must be considered that both proteins are synthesized in the liver, and their levels decrease not only in malnutrition but also in other situations (e.g., inflammation, severe liver diseases) (12).

During inflammatory processes, the synthesis of prealbumin and albumin is reduced, while the synthesis of acute phase proteins, such as CRP, which is also synthesized in the liver, is increased. Thus, in inflammation, it is desirable to assess prealbumin with respect to CRP changes. Nevertheless, in the perioperative process, when CRP increase is expected, prealbumin is a useful parameter not only for monitoring nutritional status but also for planning nutritional intervention. It has been recommended that an increase in prealbumin of more than 0.04 g/L per week is a sign of satisfactory nutritional support (13). In our patient, nutritional support resulted in a 0.17 g/L increase in prealbumin level over 3 weeks (see Table 2, H2-Day 7 and H3-Day 0), which is even higher than the recommended value. Due to successful nutritional support, the first reconstruction could be performed at the beginning of H3. Thus, according to the results obtained, prealbumin can be used for the timing of reconstruction, which is also consistent with prediction of successful surgical procedure outcomes (9, 14). Our findings reinforce the crucial importance of real-time biochemical monitoring in perioperative planning.

Another factor influencing the healing of PUs and the success of reconstructive procedures is the level of oxidative stress (15). However, not only reactive oxygen species (ROS) but also inflammation play an important role in the complex process of wound healing. ROS activate signaling pathways leading to the production of cytokines such as IL-6 and TNF-*α*, which subsequently modulate the inflammatory phase of healing (15, 16). A marker of inflammation is CRP, the level of which increases in response to cytokines (e.g., IL-6 and TNF-α). Although ROS and CRP are not directly related, they are linked through inflammatory pathways (17).

Among the biomarkers of oxidative stress, urinary 8-OHdG is important, since it reflects the level of DNA damage (4). However, it has not yet been studied in patients with PUs. Thus, this study is the first to describe the dynamics of its levels during long-term treatment of multiple PUs in a paraplegic patient. The novelty of our study lies in the integration of oxidative stress parameters, particularly 8-OHdG, as a biomarker for surgical readiness in PU patients. While previous research has focused primarily on nutritional markers such as albumin and prealbumin (9, 12, 14), our study provides new evidence linking oxidative stress with clinical outcomes in the setting of long-term complex PU treatment.

It is well known that 8-OHdG is a highly recognized parameter of oxidative stress, and high levels of 8-OHdG are a risk factor for a number of pathologies (18–21). For example, elevated levels of urinary 8-OHdG are detected in patients with carcinomas and in diabetic patients (22). Yet, in this case, we observed different results. During H1 - H3, an inverse course of 8-OHdG and CRP was observed. When CRP was elevated, a low level of 8-OHdG was measured. The association of 8-OHdG with CRP was observed in a paraplegic patient with a severe clinical condition, which precluded PU reconstruction. This was accompanied by decreased values of nutritional parameters (especially prealbumin). Decreased serum 8-OHdG values were reported in several studies focusing on various pathologies, e.g., breast cancer (23, 24), polycystic ovary syndrome (25), and colorectal cancer (26). It has been shown that the low expression of 8-OHdG is associated with poor prognosis of breast cancer (24) and colorectal cancer (26). In this study, we assume that the main reason for our findings is the patient’s overall exhaustion. Moreover, the novel relationship between oxidative stress, as represented by urinary 8-OHdG, and inflammatory markers (particularly CRP) provides new insights into the biochemical dynamics during severe clinical conditions. This inverse trend suggests a unique biochemical response influenced by the patient’s overall health and exhaustion. To our best knowledge, this has not been previously described in the context of PU treatment.

During subsequent hospitalizations, the nutritional status and insignificantly fluctuating CRP and 8-OHdG values allowed reconstructions to be performed as scheduled. The data demonstrate that progressive nutritional optimization, particularly with high-protein supplementation, played a crucial role in stabilizing the patient’s metabolic status and enabling surgical interventions. These findings further support the importance of individualized, dynamic nutritional strategies in PU treatment and surgical planning.

This long-lasting process led to healing of all PUs. The 12-month follow-up after the last hospitalization revealed satisfactory results. The dynamics of the monitored biochemical parameters in the context of treating multiple PUs proved to be crucial for the timing of surgical reconstruction and achieving satisfactory therapeutic outcomes (see Figure 1B). The case also underscores the importance of continuous patient engagement and psychological support throughout long-term reconstructive treatments. Repeated hospitalizations and surgeries can lead to psychological distress, affecting treatment adherence and overall recovery. A multidisciplinary approach, including psychological counseling, enhances patient motivation and trust in the treatment process. Providing structured psychological support can improve emotional well-being and thus contribute to better clinical outcomes.

In the routine operation of a clinical unit, the metabolic status of the patient, determined especially by nutritional (prealbumin, albumin) and inflammatory parameters (CRP), is monitored for the timing of surgical interventions. However, the state of the body in terms of oxidative stress level is also important for the success of surgical interventions and subsequent wound healing. In routine practice, this aspect has not yet been emphasized. This gap can be filled by urinary 8-OHdG, a non-invasive oxidative stress parameter reflecting DNA damage. It may serve as a valuable tool in perioperative planning, improving both patient stratification and surgical outcomes. Therefore, by monitoring urinary 8-OHdG over time, clinicians can gain an objective, dynamic understanding of a patient’s readiness for surgical intervention.

Finally, the integration of urinary 8-OHdG into clinical decision-making can enhance the assessment of metabolic stress and healing potential in patients with multiple PUs. When combined with standard nutritional and inflammatory markers, it offers a more complete picture of the patient’s status. This multidisciplinary approach supports better timing of surgical interventions and more individualized, evidence-based care for multiple PUs patients.

Next, to its contribution, this study has some limitations. Firstly, as this is a single-case study, the results may not be generalizable to a wider patient population. Secondly, although 8-OHdG was used as a highly recognized marker of oxidative stress, other oxidative and inflammatory biomarkers could provide a more comprehensive understanding of the healing process.

## Conclusion

The preparation and timing of PU reconstruction require complex approach with an emphasis on a multidisciplinary strategy. It includes local conservative treatment, surgical interventions, systemic antibiotic therapy, evaluation of the patient’s condition using laboratory parameters, and nutritional and psychological support. A comprehensive approach, involving collaboration between pre-clinical and clinical experts, leads to successful long-term therapy results. Monitoring nutritional, inflammatory, and oxidative stress parameters (such as prealbumin, CRP, and 8-OHdG) is highly beneficial and desirable for the timing of surgical strategies and can advance research in this area.

## Data Availability

The datasets generated for this case report are not publicly available due to patient confidentiality. Reasonable requests to access the data should be directed to the corresponding author.

## References

[1] Shiferaw WS Akalu TY Mulugeta H Aynalem YA. The global burden of pressure ulcers among patients with spinal cord injury: a systematic review and meta-analysis. BMC Musculoskelet Disord. (2020) 21:1–11. doi: 10.1186/s12891-020-03369-0, PMID: 32471497 PMC7260823

[2] Kruger EA Pires M Ngann Y Sterling M Rubayi S. Comprehensive management of pressure ulcers in spinal cord injury: current concepts and future trends. J Spinal Cord Med. (2013) 36:572–85. doi: 10.1179/2045772313Y.0000000093, PMID: 24090179 PMC3831318

[3] Šín P Hokynková A Nováková M Paulová H Babula P Pokorná A. *Can different type of the pressure ulcers debridement affect oxidative stress parameters?* Cesk Slov Neurol N. (2022). 85/118:S34–S37. doi: 10.48095/cccsnn2022S34

[4] Cooke MS Evans MD Herbert KE Lunec J. Urinary 8-oxo-2’-deoxyguanosine–source, significance and supplements. Free Radic Res. (2000) 32:381–97. doi: 10.1080/10715760000300391, PMID: 10766407

[5] Lipovỳ B Matejovičová M Řihová H Štikarovská D Novotná L Hlaváčová M. The application of comet assay in monitoring of the immunosuppression level in a patient with toxic epidermal necrolysis: a case report. Burns Open. (2017) 1:80–3. doi: 10.1016/j.burnso.2017.05.007

[6] Henriksen T Hillestrøm PR Poulsen HE Weimann A. Automated method for the direct analysis of 8-oxo-guanosine and 8-oxo-2′-deoxyguanosine in human urine using ultraperformance liquid chromatography and tandem mass spectrometry. Free Radic Biol Med. (2009) 47:629–35. doi: 10.1016/j.freeradbiomed.2009.06.002, PMID: 19501155

[7] Hookman P Barkin JS. *Clostridium difficile* associated infection, diarrhea and colitis. World J Gastroenterol. (2009) 15:1554–80. doi: 10.3748/wjg.15.1554 PMID: 19340897 PMC2669939

[8] Haesler E. *European pressure ulcer advisory panel (EPUAP); National Pressure Injury Advisory Panel (NPIAP); Pan Pacific pressure injury Alliance (PPPIA)*. Prevention and Treatment of Pressure Ulcers/Injuries: Clinical Practice Guideline International Guideline. (2019).

[9] Loftus TJ Brown MP Slish JH Rosenthal MD. Serum levels of prealbumin and albumin for preoperative risk stratification. Nutr Clin Pract. (2019) 34:340–8. doi: 10.1002/ncp.10271, PMID: 30908744

[10] Keller U. Nutritional laboratory markers in malnutrition. J Clin Med. (2019) 8:775. doi: 10.3390/jcm8060775, PMID: 31159248 PMC6616535

[11] Ranasinghe RN Biswas M Vincent RP. Prealbumin: the clinical utility and analytical methodologies. Ann Clin Biochem. (2022) 59:7–14. doi: 10.1177/0004563220931885, PMID: 32429677

[12] Bertholf RL. Proteins and albumin. Lab Med. (2014) 45:e25–41. doi: 10.1309/LMKRNRGW5J03APZQ

[13] Dellière S Cynober L. Is transthyretin a good marker of nutritional status? Clin Nutr. (2017) 36:364–70. doi: 10.1016/j.clnu.2016.06.004, PMID: 27381508

[14] Salvetti DJ Tempel ZJ Gandhoke GS Parry PV Grandhi RM Kanter AS. Preoperative prealbumin level as a risk factor for surgical site infection following elective spine surgery. Surg Neurol Int. (2015) 6:S500–3. doi: 10.4103/2152-7806.166893, PMID: 26605112 PMC4617027

[15] Hokynková A Babula P Pokorná A Nováková M Nártová L Šín P. Oxidative stress in wound healing – current knowledge. Ceska Slov Neurol Neurochir. (2019) 82/115:S37–9. doi: 10.14735/amcsnn2019S37

[16] Soomro S. Oxidative stress and inflammation. Open. J Immunol. (2019) 9:1–20. doi: 10.4236/oji.2019.91001, PMID: 40144829

[17] Lopes FB Sarandy MM Novaes RD Valacchi G Gonçalves RV. OxInflammatory responses in the wound healing process: a systematic review. Antioxidants. (2024) 13:823. doi: 10.3390/antiox13070823 39061892 PMC11274091

[18] Jelic MD Mandic AD Maricic SM Srdjenovic BU. Oxidative stress and its role in cancer. J Cancer Res Ther. (2021) 17:22–8. doi: 10.4103/jcrt.JCRT_862_16 33723127

[19] Di Minno A Turnu L Porro B Squellerio I Cavalca V Tremoli E. 8-Hydroxy-2-deoxyguanosine levels and cardiovascular disease: a systematic review and meta-analysis of the literature. Antioxid Redox Signal. (2016) 24:548–55. doi: 10.1089/ars.2015.6508, PMID: 26650622 PMC4827317

[20] Lindqvist D Dhabhar FS James SJ Hough CM Jain FA Bersani FS. Oxidative stress, inflammation and treatment response in major depression. Psychoneuroendocrinology. (2017) 76:197–205. doi: 10.1016/j.psyneuen.2016.11.031, PMID: 27960139 PMC5272818

[21] Goriuc A Cojocaru KA Luchian I Ursu RG Butnaru O Foia L. Using 8-Hydroxy-2′-Deoxiguanosine (8-OHdG) as a reliable biomarker for assessing periodontal disease associated with diabetes. Int J Mol Sci. (2024) 25:1425. doi: 10.3390/ijms25031425, PMID: 38338704 PMC10855048

[22] Wu LL Chiou CC Chang PY Wu JT. Urinary 8-OHdG: a marker of oxidative stress to DNA and a risk factor for cancer, atherosclerosis and diabetics. Clin Chim Acta. (2004) 339:1–9. doi: 10.1016/j.cccn.2003.09.010, PMID: 14687888

[23] Sova H Jukkola-Vuorinen A Puistola U Kauppila S Karihtala P. 8-Hydroxydeoxyguanosine: a new potential independent prognostic factor in breast cancer. Br J Cancer. (2010) 102:1018–23. doi: 10.1038/sj.bjc.6605565, PMID: 20179711 PMC2844025

[24] Qing X Shi D Lv X Wang B Chen S Shao Z. Prognostic significance of 8-hydroxy-2′-deoxyguanosine in solid tumors: a meta-analysis. BMC Cancer. (2019) 19:997. doi: 10.1186/s12885-019-6189-9 31651287 PMC6813135

[25] Sova H Morin-Papunen L Puistola U Karihtala P. Distinctively low levels of serum 8-hydroxydeoxyguanosine in women with polycystic ovary syndrome. Fertil Steril. (2010) 94:2670–3. doi: 10.1016/j.fertnstert.2010.03.049, PMID: 20430377

[26] Kang M Jeong S Park S Nam S Chung JW Kim KO. Significance of 8-OHdG expression as a predictor of survival in colorectal cancer. Cancers. (2023) 15:4613. doi: 10.3390/cancers15184613, PMID: 37760582 PMC10526191

